# Primary lung cancer in children and adolescents: Analysis of a surveillance, epidemiology, and end results database

**DOI:** 10.3389/fonc.2023.1053248

**Published:** 2023-04-05

**Authors:** Weipeng Shao, Jie Liu, Bobo Li, Xiaokang Guo, Jian Sun, Hui Li, Hongbo Guo

**Affiliations:** Department of Thoracic Surgical Ward II, Shandong Cancer Hospital and Institute, Shandong First Medical University and Shandong Academy of Medical Sciences, Jinan, Shandong, China

**Keywords:** primary lung cancer, children and adolescence, histology, treatment, SEER database

## Abstract

**Background:**

The incidence of primary lung cancer (LC) in children and adolescence was rare. We analyzed data from a SEER database to better define the incidence, clinical characters, pathology, treatment, and outcomes of rare primary malignant pulmonary tumors in childhood and adolescence.

**Methods:**

Patients were chosen from the SEER database (SEER*Stat 8.4.0 software) from 2000 to 2019 and all patients were pathologically diagnosed with primary malignant tumors of the lung and bronchus. Demographic characteristics of patients (age, gender, race, primary site, laterality, location, differentiation grade, operation methods, histology, and history of radiotherapy and chemotherapy), as well as TNM stage and survival time, were collected.

**Results:**

A total of 301 cases of children ≤19 years of age with a primary malignant pulmonary tumor were reported to the SEER database from 2000 to 2019. There were 143 men (47.5%) and 158 women (52.5%). Whites represented majority of patients (79.7%), followed by Black (13.6%) and others (6.7%). As for the primary site, the main site was the lower lobe (33.2%), followed by the upper lobe (26.9%). Most of the patients (80.4%) underwent surgery. Lobectomy (39.9%) is the main operation method. Only 28 (9.3%) patients received radiotherapy and 112 (37.2) patients received chemotherapy. Carcinoid tumor was the most common histology (29.6%), followed by pulmonary blastoma (PB) (22.3%), mucoepidermoid carcinoma (MEC) (12.3%), adenocarcinoma (10.3%), neuroendocrine tumor (NET) (5.7%), squamous cell carcinoma (SCC) (5.3%), atypical carcinoma (2.3%). The mean follow-up time was 100 months. For the entire group of children and adolescents, the 1-year OS was 89.1%, and the 3-year overall survival (OS) was 79.7%. the 5-year OS was 77.9%, the 10-year OS was 75.7%, and the 15-year OS was 73.9%. And 1-year lung cancer specificity survival (LCSS) was 89.8%, and the 3-year LCSS was 80.4%. the 5-year LCSS was 79.4%, the 10-year LCSS was 77.7%, and the 15-year LCSS was 75.9%. The OS of atypical carcinoma, carcinoid tumor, and MEC were in the top three.

**Conclusions:**

Primary LC in children and adolescent were rare and histopathological diverse. Fortunately, children and adolescents with LC had an overall favorable outcome after treatment. Histology, differentiation grade, surgery, TNM stage, and therapeutic modalities have important influence on OS. The further treatment experience of each pathological type would make better evidence-based practice possible.

## Introduction

Lung cancer (LC) is the leading cause of cancer-related mortality worldwide and is estimated to contribute to 26% of cancer deaths in men and 25% in women in 2018 (1). However, the incidence of primary LC in children and adolescents is rare, estimated to be 1 in 2 million, or 0.2% of all childhood malignancies (2). Although the disease spectrum for pediatric pulmonary tumors is identical to that of adults, the various lesions have different prevalence, rates, and outcomes (3). The pathologic spectrum seen in children and adolescents with lung or endobronchial tumors is more diverse, including neuroendocrine tumor (NET), mucoepidermoid tumors (MEC), adenocarcinoma, pulmonary blastoma (PB), squamous cell carcinoma (SCC), and sarcomas (4). Survival rates vary considerably depending on tumor type, location, and stage. At present, the pediatric literature mainly includes case reports and case series (5–7). Only two population-based studies have been published using the Surveillance, Epidemiology, and End Results (SEER) database and National Cancer Data Base (NCDB), respectively (2, 4). Therefore, we analyze data from a SEER database to better define the incidence, clinical characters, pathology, treatment, and outcomes of rare primary malignant pulmonary tumors in childhood and adolescence.

## Methods

### Ethics statement

The research data files were obtained from the SEER database using the reference number 14796-Nov2021. Because there was no access to patient identity, it was not necessary to obtain informed consent.

### Patient selection

Patients were chosen from the SEER database. (SEER*Stat 8.4.0 software) (Suggested citation for the selected database: Surveillance, Epidemiology, and End Results (SEER) Program (www.seer.cancer.gov) SEER*Stat Database: Incidence - SEER Research Plus Data, 17 Registries, Nov 2021 Sub (2000-2019) - Linked to County Attributes - Time Dependent (1990-2019) Income/Rurality, 1969-2020 Counties, National Cancer Institute, DCCPS, Surveillance Research Program, released April 2022, based on the November 2021 submission.) All patients were pathologically diagnosed with primary malignant tumors of the lung and bronchus. The inclusion codes and criteria from the SEER database were as follows: histological type (exclude lymphoma and leukemia (M9590-M9993) and Kaposi sarcoma (M9140)), and primary site (C33.9-C34.3, C34.8-C34.9).

Demographic characteristics of patients (age, gender, race, primary site, laterality, location, differentiation grade, operation methods, histology, and history of radiotherapy and chemotherapy), as well as TNM stage and survival time, were collected. Survival time was considered as the time between diagnosis and death or the last follow-up time according to the SEER program definition. Overall survival (OS) was defined as the time from diagnosis to death from any cause and patients alive were censored at the time of the last recording. Lung cancer specific survival (LCSS) was defined as the time from diagnosis to death from LC cause and patients alive were censored at the time of the last recording.

### Statistical analysis

Data were presented as frequencies (percent) or median deviation (range). Comparisons of continuous variables were performed using one-way analysis of variance (ANOVA). Chi-square bivariate analysis was used for the association of binary qualitative variables. The OS and LCSS were analyzed using the Kaplan-Meier method and the log-rank test comparing survival in two or more groups. A multivariable Cox regression analysis was performed to determine the impact of these prognostic factors on OS. A two-sided P value < 0.05 was considered statistically significant.

## Results

### Incidence, clinical characteristics, and treatment

**Figure 1:**
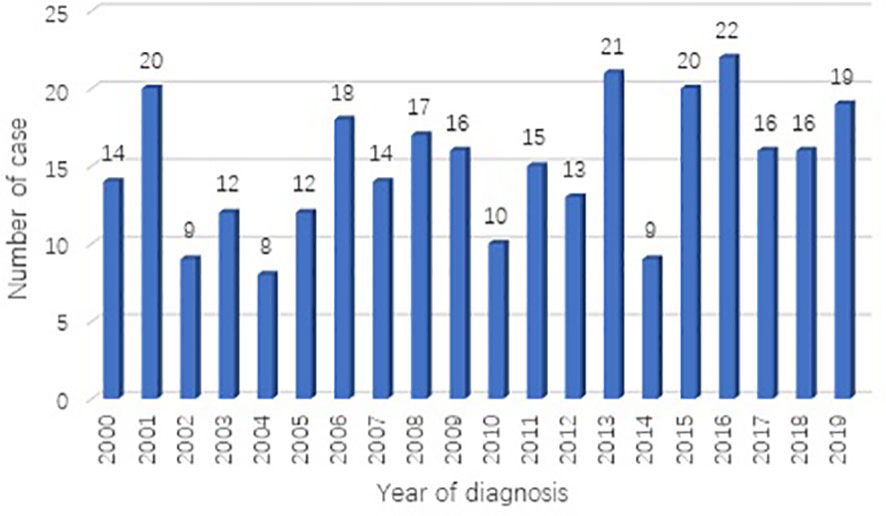
Number of primary lung cancer diagnosed each year from 2000 to 2019 in SEER database.

**Figure 2:**
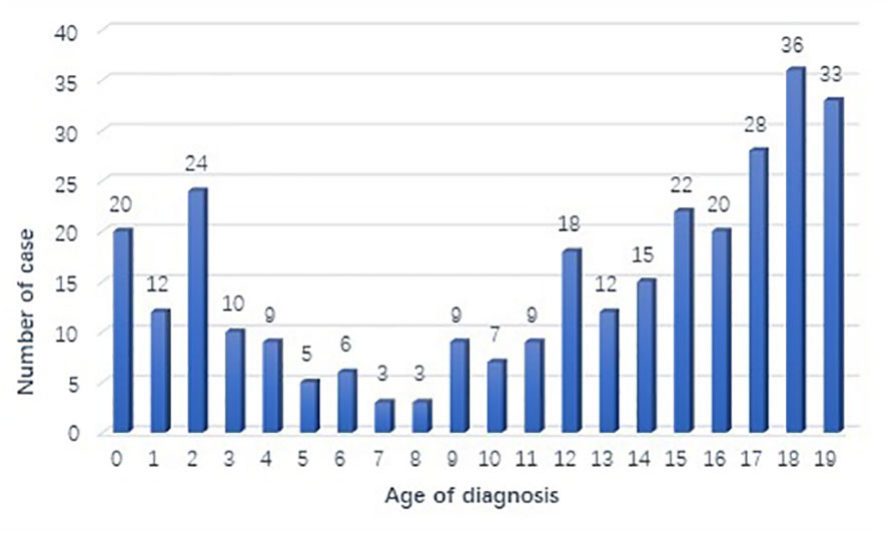
Number of primary lung cancer diagnosed each age group in SEER database.

A total of 301 cases of children ≤19 years of age with a primary LC were reported to the SEER database from 2000 to 2019. During the same period, 962, 944 LC patients were registered in SEER database. The incidence rate of LC is as low as 0.03% in children. There is no obvious regularity in the number of cases per year, about 15 cases occurred every year. The epidemic trend was shown in **Figure 1** in these years and the incidence rate of different ages was shown in **Figure 2**. The peak period of incidence was in the 0-4 age group and 15-19 age group. The demographics for the 301 patients at the presentation were described in **Table 1**. There were 143 men (47.5%) and 158 women (52.5%). Whites represented majority of patients (79.7%), followed by Black (13.6%) and others (6.7%). As for the primary site, the main site was the lower lobe (33.2%), followed by the upper lobe (26.9%). There was little lateralized specialization because the right lung accounted for 51.5% of all the study groups, and the left accounted for 42.5%. And specifically, the right lower lobe (RLL), left upper lobe (LUL), and left lower lobe (LLL) ranked in the top three respectively. Most of the patients (80.4%) underwent surgery. We found many operation methods in SEER database, including local tumor destruction (laser ablation or cryosurgery, electrocautery, and fulguration), bronchial sleeve resection, wedge resection, segmental resection, lobectomy with mediastinal lymph node dissection, Lobe or bilobectomy extended, pneumonectomy with mediastinal lymph node dissection, extended pneumonectomy plus pleura or diaphragm. Lobectomy with mediastinal lymph node dissection (39.9%) is the main operation method, followed by sublobar resection (18.6%) and pneumonectomy with mediastinal lymph node dissection (7.0%). Part of the patients did not have surgery (19.6%). Most patients did not receive surgery because they were in stage IV, but more than half of the patients did not provide complete TNM stage. Pathological differentiation degree and TNM staging were shown in **Table 1**, but the “unknow” were more than 50%. Only 28 (9.3%) patients received radiotherapy. 112 (37.2%) patients received chemotherapy.

**Table 1:** Demographics, Clinical Characteristics, and OS.

Variable	Number	Percentage	Survival Rate			λ^2^	P-value
			1-year	3-year	5-year		
Age, years						0.55	0.96
0	20	6.6	82.6	82.6	82.6		
1—4	55	18.3	89	70.7	70.7		
5—9	26	8.6	79.2	69.3	69.3		
10—14	61	20.3	96.2	75	75		
15—19	139	46.2	94.9	82.5	78.8		
Gender						0.46	0.5
Male	143	47.5	97.2	86.3	81.2		
Female	158	52.5	94.8	87.2	79.2		
Race						3.48	0.48
White	240	79.7	97.5	92.5	86.4		
Black	41	13.6	79.3	65.7	65.7		
AI	2	0.7	50	——	——		
API	15	5.0	73.3	——	——		
Unknown	3	1.0	——	——	——		
Primary site						44.5	<0.01
Upper lobe	81	26.9	94.7	84.2	78.3		
Middle lobe	27	9.0	85	85	85		
Lower lobe	100	33.2	94.9	85.9	84.1		
T and B	40	13.3	88.4	85	85		
Overlapping	16	5.3	54.5	54.5	54.5		
NOS	37	12.3	79.8	26.9	26.9		
Laterality						26.2	<0.01
Left	128	42.5	98.4	86.9	84		
Right	155	51.5	94.2	84.4	76.9		
Bilaterality	14	4.7	70.1	60.1	33.4		
NOS	4	1.32	——	——	——		
Location						60.5	<0.01
LUL	48	15.9	93.1	87.7	84.8		
LLL	43	14.3	97.5	89.8	89.8		
RUL	31	10.3	92.9	89	89		
RML	27	9.0	88.9	85	85		
RLL	57	18.9	87.4	83.1	83.1		
Trachea	4	1.3	100	100	100		
Bilaterality	7	2.3	57.1	57.1	38.1		
LM	24	8.0	95.8	87.1	87.1		
RM	12	4.0	100	88.9	77.8		
NOS	32	10.6	80.2	45.1	39.4		
overlapping	16	5.3	81.3	54.5	54.5		
Histology						110	<0.01
Carcinoid tumor	89	29.6	97.6	97.6	97.6		
MEC	37	12.3	97.1	97.1	——		
Adenocarcinoma	31	10.3	78.3	55.4	——		
SCC	12	4.0	20.8	——	——		
NET	16	5.3	48.7	——	——		
Pulmonary blastoma	67	22.3	93.7	77.2	70.5		
Atypical carcinoma	7	2.3	100	——	——		
other, NOS	42	14.0	84.9	43.4	34.7		
Grade						67.2	<0.01
I	53	17.6	96.2	96.2	——		
II	30	10.0	76.2	——	——		
III	18	6.0	36.1	11.3	——		
IV	10	3.3	77.8	44.4	44.4		
Unknown	190	63.1	97.3	93.3	85.8		
Surgery						100	<0.01
no	59	19.6	89.4	47.2	23.4		
yes	242	80.4	97.5	94.4	90.1		
operation methods						111	<0.01
Local tumor destruction or excision	9	3.0	88.9	76.2	76.2		
sublobar resection	56	18.6	98.2	94.2	89.6		
Bronchial sleeve resection	13	4.3	100	100	100		
lobectomy	120	39.9	96.6	89.8	87.2		
Pneumonectomy	21	7.0	75.4	——	——		
Lobe or bilobectomy extended	16	5.3	100	100	100		
resection NOS	7	2.3	100	40	40		
TNM stage						105	<0.01
I	65	21.6	100	97.9	97.9		
II	20	6.6	100	100	100		
III	24	8.0	59.7	——	——		
IV	33	11.0	74.6	34.4	13.1		
unknown	159	52.8	97.5	87.1	81.8		
Radiotherapy						1.43	0.23
yes	28	9.3	75	——	——		
no	273	90.7	97.8	93.2	88		
Chemotherapy						108	<0.01
yes	112	37.2	75.9	51.7	48.1		
no/unknown	189	62.8	98.9	96.9	96.1		

AI, American Indian/Alaska Native; API, Asian or Pacific Islander; T and B, Trachea and Main bronchus; Overlapping, Overlapping lesion of lung; LUL, left upper lobe; LLL, left lower lobe; RUL, right upper lobe; RML, right middle lobe; RLL, right lower lobe; LM, left main bronchus; RM, right main bronchus; MEC, mucoepidermoid carcinoma; SCC, squamous cell carcinoma; NET, neuroendocrine tumor.

As summarized in **Table 2**, there were 75 in the 0-4 age group, 26 in the 5-9 age group, 61 in the 10-14 age group, and 139 in the 15-19 age group. The incidence of pulmonary blastoma in the 0-4 age group was the highest, accounting for 78.7%; In the 5-9 age group, the incidence rate of MEC was the highest. The incidence of carcinoid tumor in the 10-14 age group was the highest, accounting for 37.7%, followed by adenocarcinoma (19.7%) and MEC (14.8%); In the 15-19 age group, the incidence rate of carcinoid tumor was the highest, accounting for 46.0%.

**Table 2:** Demographics and Clinical Characteristics by age group.

Variable	Age, years				P value
	0—4	5—9	10—14	15—19	
Gender					0.46
Male	40	10	31	62	
Female	35	16	30	77	
Race					0.6
White	62	17	46	115	
Black	7	7	9	18	
AI	0	0	1	1	
API	5	2	4	4	
Primary site					0.65
Upper lobe	25	6	16	34	
Middle lobe	5	3	4	15	
Lower lobe	24	6	18	52	
Overlapping	4	2	3	7	
T and B	6	6	9	19	
NOS	11	3	11	12	
Laterality					0.37
Right	36	12	30	77	
Left	37	11	27	53	
Bilaterality	1	3	2	8	
NOS	1	0	2	1	
Histology					<0.01
Carcinoid tumor	1	1	23	64	
MEC	3	11	9	14	
Adenocarcinoma	2	5	12	12	
SCC	0	1	1	10	
NET	0	0	2	14	
Pulmonary blastoma	59	3	3	2	
Atypical carcinoma	0	0	1	6	
other	10	5	10	17	
Grade					0.02
I	4	7	11	31	
II	2	3	10	15	
III	3	2	4	9	
IV	4	0	2	4	
Unknown	62	14	34	80	
Surgery					0.03
Yes	69	21	48	104	
No	6	5	13	35	
TNM stage					<0.01
I	4	10	21	30	
II	1	1	5	13	
III	1	3	3	17	
IV	1	2	7	23	
Unknown	68	10	25	56	
Radiotherapy					0.12
yes	6	0	4	18	
no	69	26	57	121	
Chemotherapy					<0.01
yes	50	5	13	44	
no/unknown	25	21	48	95	

AI, American Indian/Alaska Native; API, Asian or Pacific Islander; T and B, Trachea and Main bronchus; Overlapping, Overlapping lesion of lung; MEC, mucoepidermoid carcinoma; SCC, squamous cell carcinoma; NET, neuroendocrine tumor.

### Pathology

Carcinoid tumor was the most common histology (29.6%), followed by PB (22.3%), MEC (12.3%), adenocarcinoma (10.3%), NET (5.7%), SCC (5.3%), atypical carcinoma (2.3%). There are still many rare LC pathological types in children, which are not listed in **Table 3**, such as: primitive neuroectodermal tumor, fibromyxosarcoma, malignant teratoma, malignant hemangioendothelioma, neuroblastoma, nuclear protein in testis (NUT) associated carcinoma, lymphoepithelial carcinoma, desmoplastic small round cell tumor, pseudosarcomatous carcinoma, spindle cell sarcoma. We listed the biological characteristics and treatment plans (surgery (different operation methods), chemotherapy, and radiotherapy) for different pathological types in **Table 3**.

**Table 3:** Demographics and Clinical Characteristics by histopathologic tumor type.

Variable	Caicinoid	MEC	AD	SCC	NET	Blastoma	Atypical	Other	P value
	89	37	31	12	16	67	7	42	
Gender									0.64
Male	39	17	12	6	6	34	4	25	
Female	50	20	19	6	10	33	3	17	
Race									0.29
White	79	25	25	7	12	58	5	29	
Black	8	6	4	4	4	5	2	8	
AI	0	1	0	0	0	0	0	1	
API	1	5	2	1	0	0	0	1	
Primary site									<0.01
Upper	20	10	7	2	5	23	2	12	
Middle	11	6	1	2	0	6	0	1	
Lower	36	9	15	5	4	21	2	8	
Overlapping	4	1	1	1	2	3	0	4	
T and B	16	11	2	0	2	3	2	4	
NOS	2	0	5	2	3	11	1	13	
Laterality									<0.01
Right	55	21	14	6	6	30	3	20	
Left	33	15	13	3	9	36	4	15	
Bilaterality	0	0	3	3	1	1	0	6	
NOS	1	1	1	0	0	0	0	1	
Grade									<0.01
I	22	14	4	3	5	2	2	1	
II	2	15	5	2	2	1	1	2	
III	0	1	5	1	2	3	0	6	
IV	0	1	0	0	1	2	0	6	
Unknown	65	6	17	6	6	59	4	27	
Surgery									<0.01
Yes	83	27	22	5	7	63	6	19	
No	6	0	9	7	9	4	1	23	
Local tumor destruction or excision	2	1	2	0	0	2	0	2	
sublobar resection	10	2	10	1	2	26	0	5	
Bronchial sleeve resection	8	3	0	0	0	1	1	0	
lobectomy	50	20	9	3	2	25	3	8	
Pneumonectomy	7	8	1	0	1	2	1	1	
Lobe or bilobectomy extended	6	1	0	1	2	4	1	1	
resection NOS	0	2	0	0	0	3	0	2	
TNM stage									<0.01
I	24	24	9	0	3	0	5	0	
II	8	4	4	0	2	0	1	1	
III	4	3	5	5	4	1	0	2	
IV	1	0	12	7	6	0	0	7	
Unknown	52	6	1	0	1	66	1	32	
Radiotherapy									<0.01
Yes	2	2	5	3	5	7	1	3	
N0	87	35	26	9	11	60	6	39	
Chemotherapy									
yes	1	1	17	9	9	46	1	28	<0.01
no	88	36	14	3	7	21	6	14	

AI, American Indian/Alaska Native; API, Asian or Pacific Islander; T and B, Trachea and Main bronchus; Overlapping, Overlapping lesion of lung; MEC, mucoepidermoid carcinoma; SCC, squamous cell carcinoma; NET, neuroendocrine tumor.

### Survival

**Table 1** shows the 1-year, 3-year, and 5-year survival rate of various variables and their impact on OS. Primary site, laterality, location, histology, grade, surgery or not, TNM stage, and chemotherapy have significant effects on OS. The mean follow-up time was 100 months. For the entire group of children and adolescents, the 1-year OS was 89.1%, and the 3-year OS was 79.7%. the 5-year OS was 77.9%, the 10-year OS was 75.7%, and the 15-year OS was 73.9%. And 1-year LCSS was 89.8%, and the 3-year LCSS was 80.4%. the 5-year LCSS was 79.4%, the 10-year LCSS was 77.7%, and the 15-year LCSS was 75.9%. Surgery was associated with improved 5-year OS (90.1% versus 23.4% for no surgery, P<0.01). Tumor histology was also an important factor, and there were significant differences in survival rates among different types of tumors (P<0.01).

**Table 4** summarized the results of the multivariable analysis using the Cox regression model. Multivariable analysis of the entire cohort revealed that for OS, chemotherapy (P<0.01), surgery (P<0.01), tumor location(P=0.02)and grade (P=0.03) were an independent predictors of prognosis.

**Table 4:** Multivariable analysis OS.

	OR	95%CI	P
chemotherapy	0.06	0.03-0.12	<0.01
surgery	0.29	0.17-0.50	<0.01
location	1.10	1.02-1.20	0.02
grade	0.81	0.67-0.98	0.03

OS, overall survival; OR, odds ratio; CI, confidence interval.

**Figure 3:**
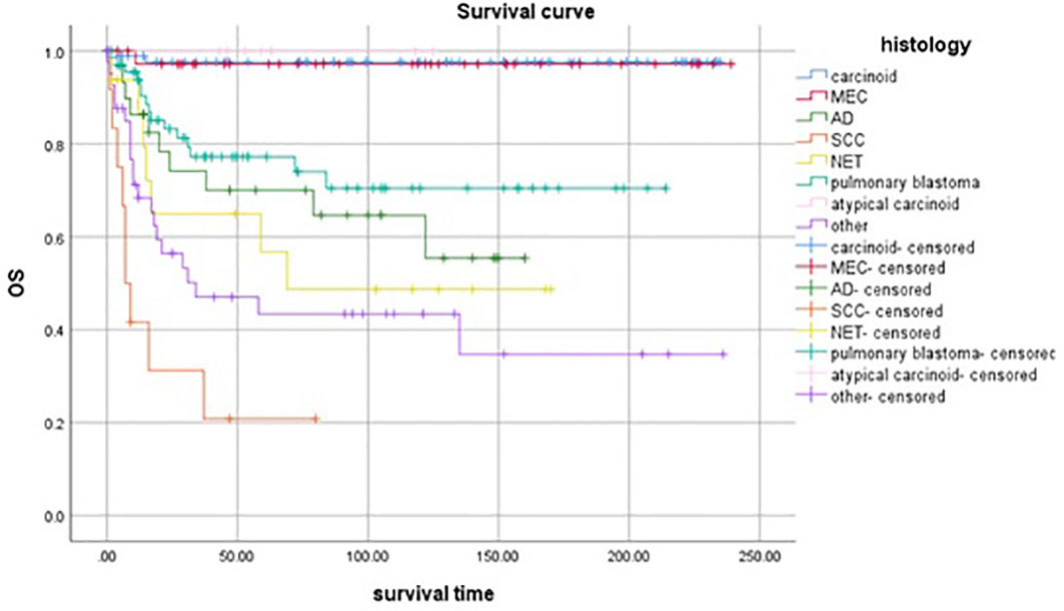
Survival curve of patients with different histological tumor types.

**Figure 3** showed the survival curve of different histological types. The OS of atypical carcinoma, carcinoid tumor, and MEC were in the top three. Patients with that had a significantly better OS compared to patients with other histology.

## Discussion

Primary LC was quite rare in children and adolescence (2). Given the low incidence, it was difficult to study the treatment and outcomes of these tumors at an institutional level. To our knowledge, the current study represents the largest (number of patients was 301) and latest (Year of diagnosis: 2000-2019) population-based study to date of primary malignant pulmonary tumors in children and adolescence.

In our research, from the 0-4 age group with the highest incidence rate of PB to the 15-19 age group with the highest incidence rate of carcinoid tumor, the histological types of lung cancer were different in different age groups. Age at presentation was one of the variables to consider when deciding the diagnostic workup. Signs and symptoms at presentation were non-specific and similar to other, more prevalent, infectious diseases or congenital lesions (8, 9). It was difficult to consider a primary LC when consulted upon a child with cough and fever, and almost impossible to do it if the patient presents with seizures. Numerous reports in the literature have suggested that primary malignant pulmonary tumors in children and adolescents present with a more aggressive disease and had a poorer prognosis than adults (10, 11). The outcome for children with pulmonary malignancies was different from adults. In adults, the 5-year OS for all patients with lung cancer was 15%. The 5-year OS rates of early-stage NSCLC patients were in the range of 40% to 70% following standard surgical treatment or stereotactic body radiation therapy (12, 13). Different from previous studies, the 5-and 10-year OS of children and adolescents in this study were 77.9% and 75.7%, respectively. In addition, we summarized the 15-year of OS and LCSS. It might be due to the different clinical and biological characteristics of different types of histology (3).

Although histologic variants of lung cancer remain identical between pediatric and adult patients, the frequency of occurrence was different (14). Carcinoid and less common subtypes of bronchogenic tumors appeared more common in the pediatric age group. SCLC and SCC common in adults, were rare (3). There are other rare case reports, such as Ewing’s Sarcoma/Primitive Neuroectodermal Tumor of the Lung (15), malignant hemangioendothelioma (16), nuclear protein in testis (NUT) (17), and so on.

NETs of the lung were classified into well-differentiated, typical, and atypical carcinoids and a poorly differentiated subgroup which includes LCLC and SCLC (18). Carcinoid tumors accounted for 2% of all lung tumors and 50% - 80% of lung cancer diagnosed in children and adolescents (3, 7, 19). In our study, it accounted for 29.6% and was the highest incidence rate of histological type. Carcinoid tumors were classified as neuroendocrine tumors, and the vast majority were characterized as typical carcinoids while the remainder were atypical carcinoids. Even though these tumors were malignant, their behavior was usually benign and was rarely responsible for the death of patients even after an extended period of follow-up. We found that it was a histological type with the best prognosis. However, LCLC and SCLC, they have the lowest incidence rate, the worst prognosis, and the lowest OS. The results were similar to the previous literature (2, 20).

MEC was defined by the World Health Organization as a tumor characterized by a combination of mucus-secreting, squamous, and intermediate cell types and was not an uncommon tumor in general (21). MEC of the lung, however, was rare with a reported frequency of 0.1% to 0.2% of primary lung tumors (22). But MEC was the second most common primary malignant epithelial pulmonary tumor seen in children (20). In this study, the rate of MEC was 12.3% and all patients received surgical treatment. The incidence was similar to that of Yesenia Rojas (4). Histologically, these tumors were divided into high and low grades based on mitotic activity and cellular differentiation. 83.3% of patients were well-differentiated (Grade I) and moderately differentiated (Grade II); There was only one poorly differentiated (Grade III) and one undifferentiated (Grade IV) case respectively. The survival time was less than 12 months. The present study explored this association and found significantly better survival in patients with histologically low-grade tumors than in patients with high-grade tumors. On the whole, the 3-year OS of MEC was excellent and in our study was 97.1%.

Adenocarcinoma was the most common type of lung cancer in adults, but the true incidence has not been established in childhood and adolescence (4, 23). Our analysis showed that adenocarcinoma represents 10.3% of cases in the study population. Neville et al. reported that the 5-year OS in their series was 26% (2). In our study, it might be the emergence of new drugs and the progress of technology that promoted the 3-year OS of adenocarcinoma to 55.4%.

SCC represents 12% of cases in children compared with 35% in the adult population (23, 24). In the study, the incidence rate was 4.0%. There were 10 (83.3%) cases in the 15-19 age group. Perhaps because of the delayed diagnosis, 7 (58.3%) patients did not receive surgical treatment. Of the lung SCC in children, metastatic tumors far exceed the number of primary lesions. Delayed diagnosis may have a role in the higher prevalence of metastatic disease at presentation (2). SCC had a poor prognosis in children and adolescents. Based on our study, the 1-year OS for SCC in pediatric patients was 20.8%.

PB was a rare malignant embryonal mesenchymal neoplasm of the lung occurring almost exclusively in children and adolescents (25). We retrieved 67 (22.3%) of these patients. There were 59 (88.1%) cases in the 0-4 age group. Surgical treatment was the primary choice for PB. In our research, 64(94.0%) patients received surgical treatment. The 5-year OS was 70.5%, which was higher than that of adenocarcinoma.

Multivariate analysis has demonstrated that chemotherapy, surgery, tumor location and grade were independent predictors for OS. But more than half of the patients did not provide complete pathological differentiation degree and TNM staging. Therefore, we believe that the positive results in the multivariate analysis do not have significance for promotion.

The advantage of this study was that the sample size was relatively large, and by extracting data from the SEER database in real-world clinical practice. Inevitably, there were some limitations in our study. Firstly, as a retrospective study, although the sample size was relatively large, we could not avoid selection bias. Furthermore, Clinical symptoms, imaging data, accurate staging, and specific treatment regime were not available in the SEER database.

## Conclusion

Primary lung cancer in children and adolescent were rare and histopathological diverse. Fortunately, children and adolescents with lung cancer had an overall favorable outcome after treatment. The further treatment experience of each pathological type would make better evidence-based practice possible.

## Data availability statement

The original contributions presented in the study are included in the article/supplementary material. Further inquiries can be directed to the corresponding author.

## Author contributions

WS, HG: research ideas and drafting drafts. WS, JL: statistical analysis. WS, BL: data extraction and manuscript writing. WS, JL, XG, JS: conception of research. WS, JS, HL, HG: review of the draft. WS, HG: quality control. All authors contributed to the article and approved the submitted version.
